# Finite Element Analysis of Dynamic Recrystallization Model and Microstructural Evolution for GCr15 Bearing Steel Warm–Hot Deformation Process

**DOI:** 10.3390/ma16134806

**Published:** 2023-07-04

**Authors:** Xuewen Chen, Jiawei Sun, Yisi Yang, Bingqi Liu, Yahui Si, Junzhuo Zhou

**Affiliations:** School of Materials Science and Engineering, Henan University of Science and Technology, 263 Kaiyuan Avenue, Luoyang 471023, China; 210321020208@stu.haust.edu.cn (J.S.); 15838510562@163.com (Y.Y.); lbq8565@stu.haust.edu.cn (B.L.); siyahui@stu.haust.edu.cn (Y.S.); 191402060632@stu.haust.edu.cn (J.Z.)

**Keywords:** GCr15 bearing steel, warm–hot deformation, dynamic recrystallization model, finite element method (FEM)

## Abstract

Warm deformation is a plastic-forming process that differs from traditional cold and hot forming techniques. At the macro level, it can effectively reduce the problem of high deformation resistance in cold deformation and improve the surface decarburization issues during the hot deformation process. Microscopically, it has significant advantages in controlling product structure, refining grain size, and enhancing product mechanical properties. The Gleeble-1500D thermal–mechanical physical simulation system was used to conduct isothermal compression tests on GCr15 bearing steel. The tests were conducted at temperatures of 600–1050 °C and strain rates of 0.01–5 s^−1^. Based on the experimental data, the critical strain model and dynamic recrystallization model for the warm–hot forming of GCr15 bearing steel were established in this paper. The model accuracy is evaluated using statistical indicators such as the correlation coefficient (R). The dynamic recrystallization model exhibits high predictive accuracy, as indicated by an R-value of 0.986. The established dynamic recrystallization model for GCr15 bearing steel was integrated into the Forge^®^ 3.2 numerical simulation software through secondary program development to simulate the compression process of GCr15 warm–hot forming. The dynamic recrystallization fraction was analyzed in various deformation regions. The grain size of the severe deformation zone, small deformation zone, and difficult deformation zone was compared based on simulated compression specimens under the conditions of 1050 °C and 0.1 s^−1^ with the corresponding grain size obtained with measurement based on metallographic photos; the relative error between the two is 5.75%. This verifies the accuracy of the established dynamic recrystallization and critical strain models for warm–hot deformation of GCr15 bearing steel. These models provide a theoretical basis for the finite element method analysis and microstructure control of the warm–hot forming process in bearing races.

## 1. Introduction

The ring rolling process is widely used to manufacture various seamless rings, especially in the production of bearing rings [1]. Ring rolling is a continuous local plastic deformation process in which the diameter of a blank is gradually expanded within a hole formed by extrusion rolls and a mandrel, decreasing the ring’s thickness while shaping its cross-sectional profile [2,3]. Based on the deformation temperature, the traditional ring rolling method can be divided into three types: hot, cold, and warm. Hot ring rolling can reduce the deformation resistance during the deformation process. However, it can also lead to surface decarburization of the workpiece, larger cutting allowances, and increased energy consumption and production costs. The cold rolling process involves high deformation resistance, which demands advanced performance from rolling equipment [4,5]. Warm ring rolling can address issues such as the excessive deformation resistance force in cold forming and the inability to manufacture complex shapes. It can also improve, to some extent, the surface decarburization and low surface dimensional accuracy defects encountered in the current hot working process of bearing steel material [6]. On the other hand, the warm rolling process is of great significance in avoiding rapid growth of the microstructure, obtaining smaller grain size, and improving the mechanical properties of bearing rings. K. Ryttberg et al. [7] investigated the changes in microstructure and texture of initial rectangular section rings made of 100Cr6 steel during the cold rolling process. Zhihao Feng et al. [8] extensively studied the impact of reduction on microstructure evolution and mechanical properties of near α-Zr alloy during the cold rolling process. Yi Yao et al. [2] clarified the correlation mechanism between deformation and strain level in hot-rolled Mg–Gd–Y–Zr alloy rings by analyzing the heterogeneity of the microstructure and texture. Dongsheng Qian et al. [1] proposed a method for achieving microstructure spheroidization directly by warm rolling 52,100 bearing steel based on the beneficial effects of eutectoid transformation and plastic deformation and verified its feasibility through experimental research.

GCr15 bearing rings typically operate under alternating tensile and compressive loads, and the microstructure and properties of the material can significantly impact its reliability and service life. Achieving a finer grain structure is critical to enhancing the material’s mechanical properties and service life [9,10,11]. It is well known that the crystal structure undergoes dynamic recovery (DRV) and dynamic recrystallization (DRX) during the deformation process at a specific process condition. The microstructural quality of the alloy is determined by the interplay between these two mechanisms [12,13]; utilizing dynamic recrystallization can result in finer grains in the alloy. Beibei Dong et al. [14] researched the dynamic recrystallization behavior of Mg–Gd–Y–Zn–Zr alloy during multi-directional forging as well as the grain refinement strengthening mechanism during severe plastic deformation. Bingtao Tang et al. [15] studied the toughness/semi-cleavage fracture and the effect of dynamic recrystallization (DRX) on grain size evolution in A7075 alloy during the process of hot stamping. Yuxiang Han et al. [16] studied the microstructure and mechanical properties of Mg97Y2Zn1 alloy during rolling at 450 °C and 500 °C. They found that more dynamic recrystallization and refined DRX grains improved the alloy’s ultimate strength and ductility.

The evolution of dynamic recrystallization is a dynamic process related to processing parameters. However, observing the dynamic evolution of the microstructure is extremely difficult. The numerical simulation gives an efficient approach to observing the evolution of the microstructure in GCr15 bearing steel [17]. The finite element method (FEM) is a modern computational technique that utilizes the principles of mechanics and provides an efficient and cost-effective approach to simulating various material processing procedures. Thomas Josef Baron et al. [9] provided an approach for describing the flow behavior of metallurgical materials using the FEM. They used this method to simulate the dynamic evolution process of the microstructure of high-strength martensitic steel MS-W1200 in a hot compression test. Chun-Nan Lin et al. [18] used a combination of a three-dimensional processing map and finite element numerical simulations to optimize the hot forming processing parameters of extruding 7005 alloy. Chong-Xiang Yue et al. [11] investigated the DRX behavior of GCr15 bearing steel under different temperatures and strain rates (950–1150 °C, 0.1–10 s^−1^). They developed a dynamic recrystallization kinetics model based on an approximate stress–strain curve model. However, they did not combine their established dynamic recrystallization model with numerical simulation technology.

At the moment, there are limited studies on the microstructure evolution and dynamic recrystallization model and microstructure numerical simulation of GCr15 bearing steel during warm forming.

It is crucial to study the microstructure evolution of bearing rings and promote dynamic recrystallization degree during the forming process, which significantly improves the mechanical performance of bearing rings, such as strength, plastic toughness, and prolonging service life. Consequently, the GCr15′s characteristics of the microstructural evolution during warm–hot forming were studied in this article using numerical simulation techniques. The aim is to establish a dynamic recrystallization kinetics model for GCr15 that can effectively characterize the recrystallization evolution of the forming process. The specimens were undergoing compression tests using a Gleeble-1500D thermal–mechanical physical simulation system under varying temperatures and strain rates (600–1050 °C, 0.01–5 s^−1^). The obtained metallographic photographs and rheological stress data from the compressed specimens were analyzed to investigate the microstructural evolution of GCr15. The accuracy of the dynamic recrystallization model for GCr15 was validated by comparing the grain size results obtained from finite element simulation with the actual grain size measurements from metallographic photographs. This provides a theoretical basis for predicting and controlling the grain size of bearing races during the microstructural evolution process in actual GCr15 bearing steel during warm–hot deformation.

## 2. Materials and Methods

Table 1 displays the elemental makeup of the GCr15 bearing steel used in the experiment. The test was carried out in accordance with the ASTM E209 standard [19]. The alloy was fashioned into cylindrical specimens measuring Φ8 mm × 12 mm, which were subsequently subjected to uniaxial compression via the employment of the Gleeble-1500D thermal–mechanical physical simulation system. The compression tests were conducted under varying temperatures and strain rates (600–1050 °C, 0.01–5 s^−1^) with a maximum deformation of 50%.

**Figure 1:**
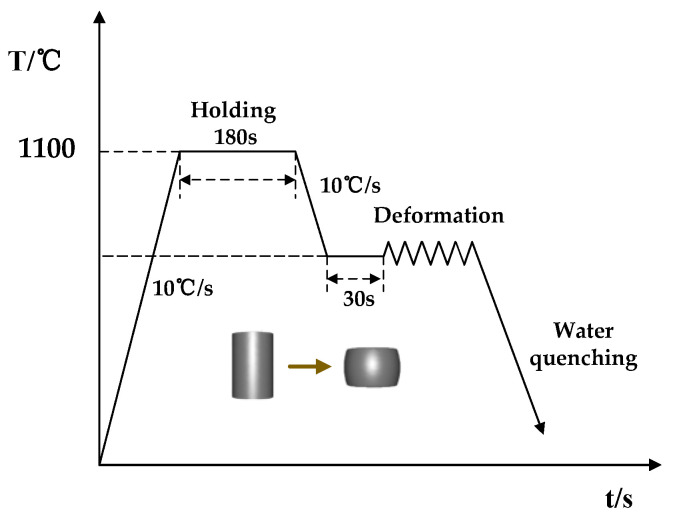
Deformation process of isothermal compression.

As illustrated in Figure 1, the specimens were heated at a temperature ramp of 10 °C per second until reaching 1100 °C, where they were held for 180 s to achieve uniform austenitization of the alloy. Subsequently, the specimens were cooled down to the target temperature and maintained for 30 s to achieve homogenized temperature distribution. Uniaxial compression was then performed to the desired reduction followed by rapid quenching of the specimen in water to preserve the microstructure after deformation.

To analyze the microstructure of the compressed sample, it was axially cut and polished with varying grades of sandpaper and the metallographic polishing machine. After polishing, the sample was etched with picric acid and dodecylbenzene sulfonic acid aqueous solution and heated in a water bath at 80 °C for 3–5 min. After corrosion completion, the sample was cleaned, dried, and observed under an electron microscope to examine its microstructure.

## 3. Results and Discussion

### 3.1. Flow Stress Curve

**Figure 2:**
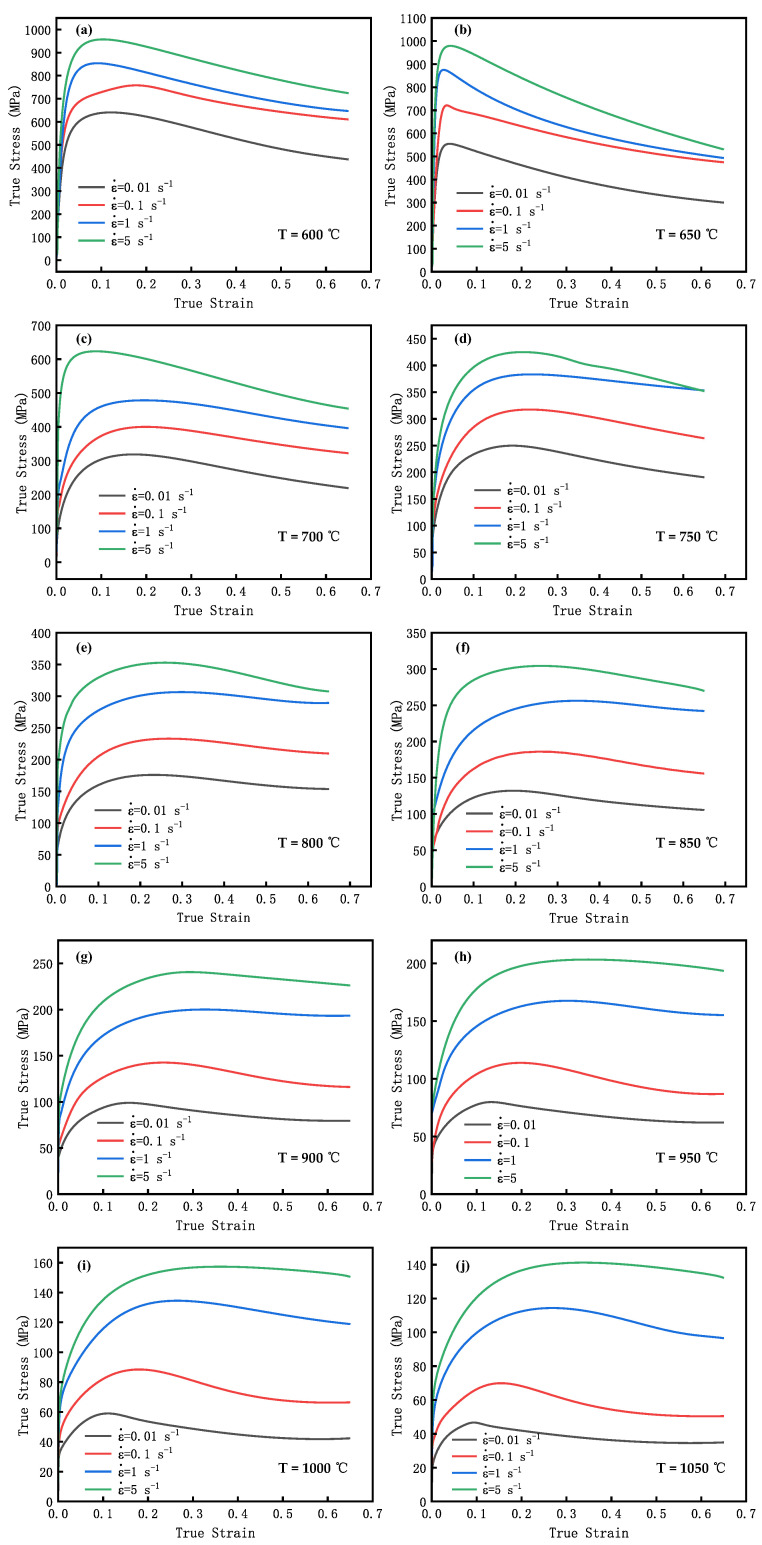
True stress–strain curves of GCr15 steel under different temperatures and strain rates. (**a**) T = 600 °C, (**b**) T = 650 °C, (**c**) T = 700 °C, (**d**) T = 750 °C, (**e**) T = 800 °C, (**f**) T = 850 °C, (**g**) T = 900 °C, (**h**) T = 950 °C, (**i**) T = 1000 °C, (**j**) T = 1050 °C.

Figure 2 displays the GCr15′s flow stress curve. These curves were derived by processing the experimental data obtained from the thermal compression test.

Figure 2h depicts the correlation between true stress and true strain of GCr15 at 950 °C and 0.1 s^−1^. In the initial stage of the compression test, the work hardening caused by the specimen’s deformation increases the true stress value sharply; upon reaching the critical strain, the true stress growth rate slows down, which can be attributed to the effects of the DRV and DRX mechanisms. As the deformation process continues, the dislocation density rapidly increases, leading to the further accumulation of strain energy. This increases the recrystallization driving force and deformation resistance, resulting in the peak stress at a true stress value of 113.812 MPa. With increasing true strain, there is a gradual decline in true stress. After a certain degree of deformation, DRX and DRV dominate, reducing the dislocation density and true stress values. With the further increase in true strain, the strengthening effect of work hardening and the softening effect of DRV and DRX tend towards dynamic equilibrium, stabilizing the true stress at 86.73 MPa.

By observing Figure 2f–h, it is found that, with a constant equivalent strain rate, the flow stress value is inversely proportional to the temperature of the compression test. At 900 °C, the peak stresses of the compression specimens (0.001 s^−1^, 0.1 s^−1^, 1 s^−1^) are 98.943, 142.554, and 200.059 MPa, respectively. As the temperature increases, the level of thermal activation within the material becomes stronger, leading to an increase in the atomic diffusion rate. This results in more dislocations undergoing climb and cross-slip, and the number of slip systems increases, further enhancing the GCr15 steel’s ability to undergo plastic deformation, ultimately causing a decrease in the true stress values. On the other hand, recrystallization is predominantly a process of nucleation and grain growth. With increasing temperature, the driving force for dynamic recrystallization is enhanced.

As shown in Figure 2g, at a constant deformation temperature, the true stress value is directly proportional to the strain rate. Elevating the equivalent strain rate makes the effect of work hardening more significant. The greater the equivalent strain rate, the shorter the DRV and DRX times and the greater the true stress value.

All specimens experienced varying degrees of DRX softening under different conditions. The true stress values decreased after reaching their peak stress. Figure 2h shows that the specimen (900 °C, 0.1 s^−1^) exhibited a higher degree of recrystallization than the specimen with the 5 s^−1^ strain rate. By observing Figure 2e,g,i, it is evident that there was an increase in the degree of dynamic recrystallization of the specimens as the temperature increased at the strain rate of 0.1 s^−1^. The experimental results demonstrated that decreasing the strain rate and elevating the processing temperature facilitate dynamic recrystallization.

### 3.2. Hansel–Spittel Constitutive Equation for GCr15 Bearing Steel

The “Hansel–Spittel” [20,21] constitutive equation is temperature-dependent and considers strain hardening or softening phenomena. It also takes the strain rate of the material into account. On the other hand, the model has a simple form and is frequently employed to characterize viscoplastic behavior. Additionally, it can be easily implemented in finite element software, such as Forge^®^, making it a popular choice for engineers and researchers in the field.

(1) $$\sigma =Ae^{m_{1}T}\varepsilon^{m_{2}}{\dot{\varepsilon }}^{m_{3}}e^{\frac{m_{4}}{\varepsilon }}{\left(1+\varepsilon \right)}^{m_{5}T}e^{m_{7}\varepsilon }{\dot{\varepsilon }}^{m_{8}T}T^{m_{9}}$$

where *m*_1_ and *m*_9_ define the material’s sensitivity to temperature, *m*_5_ term coupling temperature and strain, and *m*_8_ term coupling temperature and strain rate. *m*_2_, *m*_4_, and *m*_7_ define the material’s sensitivity to strain, *m*_3_ depends on the material’s sensitivity to the strain rate, ε is the equivalent strain, $\dot{\varepsilon }$ is the equivalent strain rate, σ is the equivalent stress, and *T* is the temperature given in Celsius.

The model’s parameters were calculated using the averaging method [22,23,24]. After performing the calculations, the material parameters associated with the constitutive model are presented in Table 2.

The constitutive equation for GCr15 can be expressed as:

(2) $$\begin{array}{ll} \sigma = & 4.16\times 10^{6}\cdot e^{-0.00463T}\cdot \varepsilon^{0.1382}\cdot {\dot{\varepsilon }}^{-0.093}\cdot e^{\frac{0.00209}{\varepsilon }}\\ & \cdot {\left(1+\varepsilon \right)}^{0.00317T}\cdot e^{-2.0621\varepsilon }\cdot {\dot{\varepsilon }}^{2.66\times 10^{-4}T}\cdot T^{-0.8565} \end{array}$$

## 4. Establishment of Dynamic Recrystallization Model for GCr15 Bearing Steel Warm–Hot Forming Process

Dynamic recrystallization refers to the procedure of grain nucleation and growth during metal deformation. Studying the microstructure evolution mechanism of DRX in GCr15 bearing steel during warm–hot deformation is crucial for analyzing its processability. Proposing an accurate theoretical model to describe the dynamic recrystallization process during the warm–hot forming process is essential for enhancing the precision of finite element simulation and predicting the microstructure evolution during deformation processes.

### 4.1. Critical Strain Model for GCr15 Bearing Steel Warm–Hot Forming Process

As illustrated in Figure 2, the curve’s maximum true stress results from the work hardening and the softening effect of DRX and DRV. The DRX is triggered when the true strain value reaches a critical strain. Generally, DRX takes place before the maximum value of flow stress is attained, and there exists a linear relationship between $\varepsilon_{c}$ and $\varepsilon_{p}$.

The correlation between the critical strain and the peak strain has been demonstrated through studies [25]:

(3) $$\varepsilon_{c}=a\cdot \varepsilon_{p}$$

Poliak [26] proposed that the DRX mechanism is associated with the θ-σ curve. When the curve displays an inflection point, dynamic recrystallization occurs. At this specific point, the flow stress value is the critical stress. This critical stress can be utilized to calculate the corresponding critical strain.

The inflection point is the point of $\partial^{2}\theta /\partial \sigma^{2}=0$, and since θ=∂σ/∂ε, so it follows that:

(4) $$\frac{\partial \theta }{\partial \sigma }=\frac{\partial \theta }{\partial \varepsilon }\times \frac{\partial \varepsilon }{\partial \sigma }=\frac{1}{\theta }\times \frac{\partial \theta }{\partial \varepsilon }=\frac{\partial \left(\ln\theta \right)}{\partial \varepsilon }$$

Therefore, the lnθ-ε curves can be plotted for all conditions, and a cubic polynomial can be performed on the curve. Subsequently, the fitted function can be differentiated.

(5) $$\ln\theta =A+B\varepsilon +C\varepsilon^{2}+D\varepsilon^{3}$$

(6) $$-\partial \left(\ln\theta \right)/\partial \varepsilon =B+2C\varepsilon +3D\varepsilon^{2}$$

The extreme value point is the critical strain point:

(7) $$\varepsilon_{c}=-\frac{2C}{2\times 3D}=-\frac{C}{3D}$$

**Figure 3:**
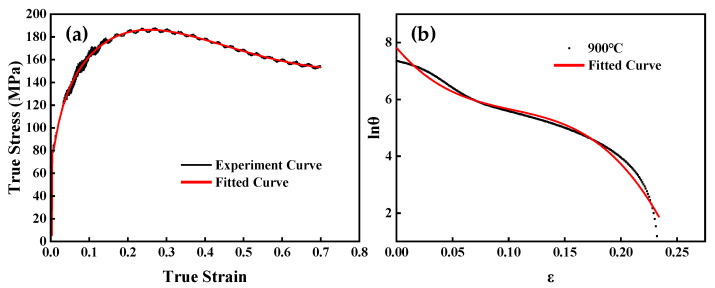
(**a**) Experimental curve and the fitted curve at the temperature of 850 °C and 0.1 s^−1^; (**b**) Relationships between lnθ and ε at 0.1 s^−1^ and 900 °C.

Figure 3a depicts the fitting of the true stress–true strain curve using Equation (8).

(8) $$\sigma =\frac{\left(a_{0}+a_{1}\varepsilon +a_{2}\varepsilon^{2}+a_{3}\varepsilon^{3}+a_{4}\varepsilon^{4}+a_{5}\varepsilon^{5}\right)}{\left(b_{0}+b_{1}\varepsilon +b_{2}\varepsilon^{2}+b_{3}\varepsilon^{3}+b_{4}\varepsilon^{4}+b_{5}\varepsilon^{5}+b_{6}\varepsilon^{6}\right)}$$

**Figure 4:**
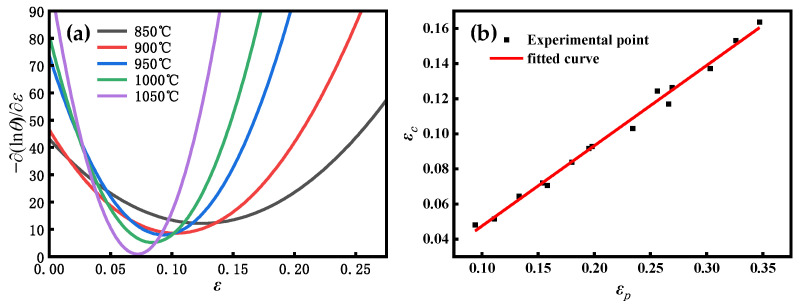
(**a**) Correlation between $-\partial \left(\ln\theta \right)/\partial \varepsilon$ and ε at 0.1 s^–1^ and different temperatures. (**b**) Correlation between $\varepsilon_{c}$ and $\sigma_{c}$.

The lnθ-ε curve is plotted. The lnθ-ε curve is fitted with the cubic equation in Origin^®^, the result is shown in Equation (9), and the fitted curve is shown in Figure 4b.

(9) $$\ln\theta =7.82255-46.41145\varepsilon +366.51687\varepsilon^{2}-1184.33308\varepsilon^{3}$$

The parameters of the lnθ-ε fitted curves at different conditions and $\varepsilon_{c}$ are listed in Table 3.

A linear regression of the critical and peak strains listed in Table 3 was performed, and the results are shown in Figure 4b. It can be calculated that $\varepsilon_{c}=0.46462\varepsilon_{p}$.

The peak strain can be calculated by using the Equation (10):

(10) $$\varepsilon_{p}=A_{p}\times d_{0}{}^{n}{\dot{\varepsilon }}^{m_{p}}\exp\left(Q_{p}/RT\right)$$

where $Q_{p}$ is the thermal activation energy. $A_{p}$, *n*, and $m_{p}$ are material-dependent constants, and *R* is the gas constant. *T* is the absolute temperature. $\dot{\varepsilon }$ is the equivalent strain rate.

Since the effect of the initial grain size on DRX is not investigated, it is assumed that the grain size is equal before deformation, i.e., it is constant, and Equation (10) can be simplified as:

(11) $$\varepsilon_{p}=A_{p}{\dot{\varepsilon }}^{m_{p}}\exp\left(Q_{p}/RT\right)$$

Taking the logarithm of the Equation (11):

(12) $$\ln\varepsilon_{p}=\ln A_{p}+m_{p}\ln\dot{\varepsilon }+Q_{p}/RT$$

When the temperature is constant, $\ln A_{p}+Q_{p}/RT$ is constant, and $m_{p}$ can be obtained from $\frac{\partial \ln\varepsilon_{p}}{\partial \ln\dot{\varepsilon }}$.

**Figure 5:**
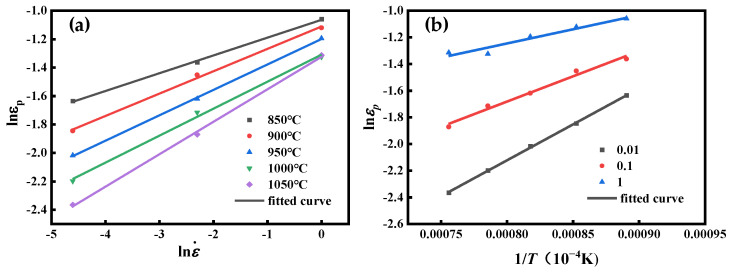
(**a**) Relationship between $\ln\varepsilon_{p}$ and $\ln\dot{\varepsilon }$. (**b**) Correlation between $\ln\varepsilon_{p}$ and 1/*T*.

The linear regression of the $\ln\varepsilon_{p}-\ln\dot{\varepsilon }$ by least squares is shown in Figure 5a, and the line’s slope is $m_{p}$. Calculate the average value of $m_{p}$ at different temperatures and the obtained $m_{p}$ is 0.175864.

When the strain rate is constant, $\ln A_{p}+m_{p}\ln\dot{\varepsilon }$ is a constant value, and the value of $A_{p}$ can be reduced to:

(13) $$\frac{Q_{p}}{R}=\frac{\partial \ln\varepsilon_{p}}{\partial 1/T}$$

**Figure 6:**
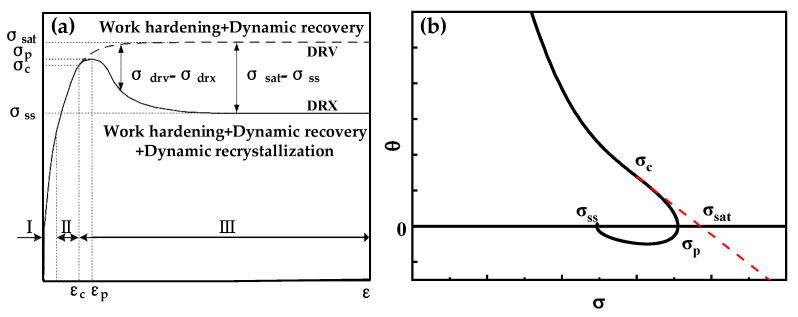
(**a**) Typical true stress–strain curve. (**b**) Typical θ-σ curve.

Linear regression was performed with least squares for $\ln\varepsilon_{p}-1/T$. The results are shown in Figure 6b. $Q_{p}/R$ can be defined by calculating the slope of the line. Further, the value of $Q_{p}$ is 31317.25019 J/mol. Thus, we can obtain the value of $A_{p}$ as 0.01371563.

The peak strain equation is:

(14) $$\varepsilon_{p}=0.013715630033339\cdot {\dot{\varepsilon }}^{0.175864}\cdot \exp\left(31317.25/RT\right)$$

The critical strain equation is:

(15) $$\varepsilon_{c}=0.006373\times {\dot{\varepsilon }}^{0.175864}\exp\left(3137.25/RT\right)$$

### 4.2. Development of DRX Model for GCr15 Bearing Steel Warm Forming Process

The Kopp model [27,28,29] is widely used to describe the dynamic recrystallization kinetic:

(16) $$\left\{\begin{array}{l} X_{\mathrm{drx}}=1-\exp\left[-k_{d}{\left(\frac{\varepsilon -\varepsilon_{c}}{\varepsilon_{0.5}-\varepsilon_{c}}\right)}^{n_{\mathrm{drx}}}\right]\\ \varepsilon_{0.5}=k_{1}Z^{n_{1}} \end{array}\right.$$

where $k_{d}$*,* $k_{1}$*,* $n_{1}$*,* and $n_{\mathrm{drx}}$ are constants, and $\varepsilon_{0.5}$ is the strain value at which the percentage of dynamic recrystallization fraction reaches 50%. ε is the equivalent strain.

#### 4.2.1. Calculation of the DRX Fraction for GCr15 Bearing Steel Warm–Hot Forming Process

**Figure 7:**
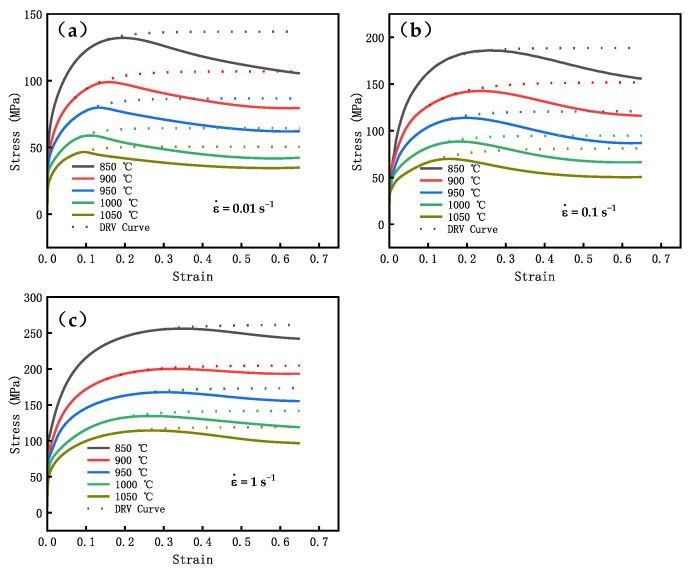
DRX curve and DRV curve at different conditions: (**a**) $\dot{\varepsilon }=\;0.01\;s^{-1}$, (**b**) $\dot{\varepsilon }=\;0.1\;s^{-1}$, (**c**) $\dot{\varepsilon }={\;1\;s}^{-1}$.

Figure 7a shows the typical stress–strain curves, where the solid line is the dynamic recrystallization (DRX) type curve, and the dashed line is the dynamic reversion (DRV) type flow stress curve.

Laasraoui et al. [30,31] found that the relationship between $X_{\mathrm{drx}}$ and the equivalent stress during the deformation of metallic materials can be expressed as:

(17) $$X_{\mathrm{drx}}=\frac{\sigma_{\mathrm{drx}}-\sigma }{\sigma_{\mathrm{sat}}-\sigma_{\mathrm{ss}}}$$

As shown in Figure 6b, the dynamic recrystallization critical point can be identified as the inflection point on the θ-σ curve. Make a tangent line from this inflection point, and the point where the tangent line intersects the line with the work-hardening rate value of zero is the saturation stress point [18]. Table 4 contains the saturation stress values of the DRV curves under different deformation conditions.

When the stress value is between the critical stress and the saturation stress, the DRV curve can be expressed as:

(18) $$\theta =m_{0}\sigma_{\mathrm{drx}}+C$$

where *m*_0_ and *C* are constants; the value of *θ* can be expressed as θ=∂σ/∂ε. The boundary condition can be derived from Figure 3a: when the value of *θ* is $\theta_{c}$, the value of $\sigma_{\mathrm{drx}}$ can be calculated as $\sigma_{c}$; when the value of *θ* is 0, the value of $\sigma_{\mathrm{drx}}$ can be calculated as $\sigma_{\mathrm{sat}}$. Substituting the boundary conditions into Equation (18):

(19) $$m_{0}=\frac{\theta_{c}}{\sigma_{c}-\sigma_{\mathrm{sat}}}$$

(20) $$C=\frac{\theta_{c}\sigma_{\mathrm{sat}}}{\sigma_{c}-\sigma_{\mathrm{sat}}}$$

(21) $$\theta =\frac{\partial \sigma }{\partial \varepsilon }=\frac{\theta_{c}\left(\sigma_{\mathrm{drv}}-\sigma_{\mathrm{sat}}\right)}{\sigma_{c}-\sigma_{\mathrm{sat}}}$$

Integrating Equation (21), the boundary condition can be derived from Figure 4a as follows: when the value of ε is $\varepsilon_{c}$, the value of σ can be calculated as $\sigma_{c}$, and we can obtain:

(22) $$\varepsilon =\varepsilon_{c}+\frac{\sigma_{c}-\sigma_{\mathrm{sat}}}{\theta_{c}}\ln(\frac{\sigma_{\mathrm{drv}}-\sigma_{\mathrm{sat}}}{\sigma_{c}-\sigma_{\mathrm{sat}}})$$

After simplification, the dynamic recovery curve of the material during deformation is changed to Equation (23):

(23) $$\sigma_{\mathrm{drv}}=\sigma_{\mathrm{sat}}+(\sigma_{c}-\sigma_{\mathrm{sat}})\exp\left[\frac{(\varepsilon -\varepsilon_{c})\theta_{c}}{\sigma_{c}-\sigma_{\mathrm{sat}}}\right]$$

The DRV curve can be obtained by substituting the values of $\sigma_{\mathrm{sat}}$, $\sigma_{c}$, $\varepsilon_{c}$, and $\theta_{c}$ under different deformation conditions into Equation (23), as shown in Figure 7.

**Figure 8:**
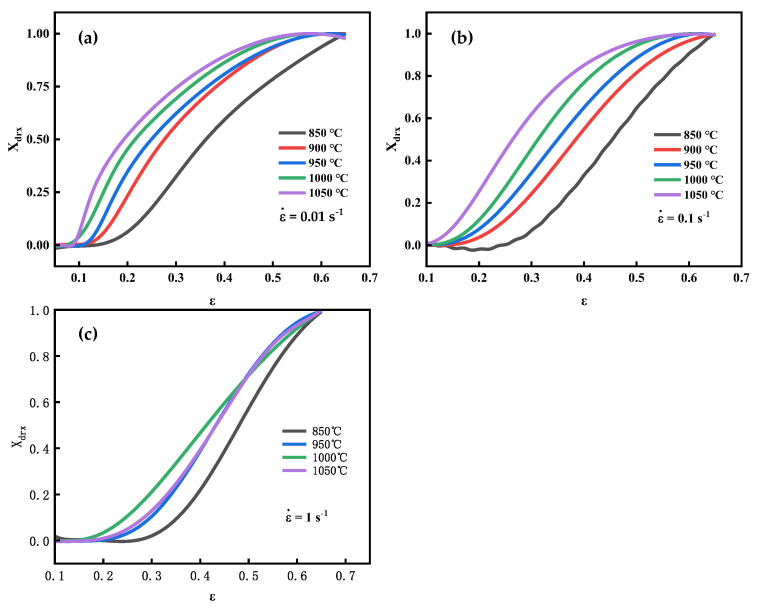
Experimental data of dynamic recrystallization fraction: (**a**) $\dot{\varepsilon }=\;0.01\;s^{-1}$, (**b**) $\dot{\varepsilon }=\;0.1\;s^{-1}$, (**c**) $\dot{\varepsilon }={\;1\;s}^{-1}$.

The calculated dynamic recrystallization fraction under different deformation conditions is shown in Figure 8. When the strain elevates, the DRX fraction of GCr15 gradually increases, and the trend of the dynamic recrystallization fraction increase is roughly the same under different conditions.

#### 4.2.2. Analysis of the Evolution of the Recrystallization Structure of GCr15 Steel

**Figure 9:**
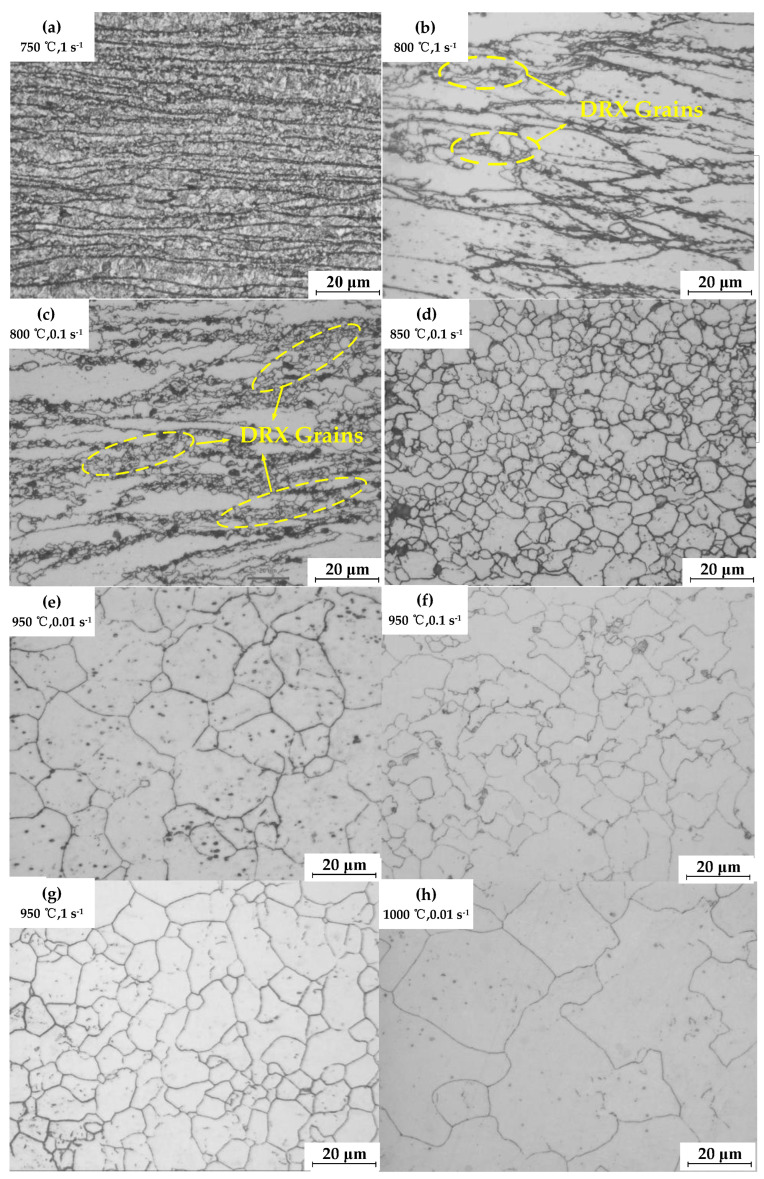
Microstructure evolution of dynamic recrystallization. (**a**) 750 °C, 1 s^−1^; (**b**) 800 °C, 1 s^−1^; (**c**) 800 °C, 0.1 s^−1^; (**d**) 850 °C, 0.1 s^−1^; (**e**) 950 °C, 0.01 s^−1^; (**f**) 950 °C, 0.1 s^−1^; (**g**) 950 °C, 1 s^−1^; (**h**)1000 °C, 0.01 s^−1^.

Figure 9a shows the compression specimens’ metallographic structure at 750 °C and 1 s^−1^. The figure shows numerous elongated and coarse-banded structures resulting from deformation. This indicates a high strain rate and a low degree of dynamic recrystallization. Figure 9b is the metallographic image at 800 °C and strain rate 1 s^−1^. With the increase in temperature, the degree of recrystallization increases, and some grains undergo the initial stage of dynamic recrystallization. The original grains’ grain borders are then invaded by new grains, forming a classic necklace-like arrangement. Figure 9c is the metallographic image at 800 °C and 0.1 s^−1^. Compared to Figure 3a, the degree of dynamic recrystallization is greater at this stage. Nonetheless, numerous small grains are formed by recrystallization, and some original grains remain unbroken, leading to a lower level of uniformity in the grain structure. Figure 9d depicts a metallographic photograph taken at 850 °C and a strain rate of 0.1 s^−1^. With increasing temperature, the driving force for recrystallization also increases, resulting in sufficient dynamic recrystallization. The grains exhibit a uniform size and are equiaxed in shape. Figure 9e–h shows that the grain size under a lower strain rate is larger than that under a higher strain rate. This phenomenon occurs because, at higher strain rates, fast accumulation of dislocations suppresses the nucleation of dynamic recrystallization, and the original grains that have not been consumed by dynamic recrystallization are retained in the initial structure, resulting in a decreased proportion of recrystallized grains. On the other hand, as the temperature increases, grain growth becomes more evident.

#### 4.2.3. Dynamic Recrystallization Characteristic Strain Determination for GCr15 Steel Temperature Forming Process

The dynamic recrystallization characteristic strain is the strain at which dynamic recrystallization occurs in 50% of the internal volume of the material. The characteristic strain value of GCr15 steel can be obtained from Figure 9.

Table 5 lists the characteristic strain values under different conditions.

The *Z* parameter [32] can be represented by Equation (24).

(24) $$Z=\dot{\varepsilon }\exp(Q/RT)$$

where *Q* is the thermal activation energy, *R* is the gas constant, and *T* is the absolute temperature. $\dot{\varepsilon }$ is the equivalent strain rate.

For the convenience of calculation, the Equation (24) can be rewritten as follows:

(25) $$\varepsilon_{0.5}=A_{0.5}d_{0}^{n_{0.5}}{\dot{\varepsilon }}^{m_{0.5}}\exp(\frac{Q_{0.5}}{RT})$$

where $Q_{0.5}$ is the thermal activation energy of 50% DRX in GCr15. $A_{0.5}$, $n_{0.5}$, and $m_{0.5}$ are material dependent constants, and *R* is the gas constant. *T* is the absolute temperature.

Since the effect of the initial grain size on DRX is not investigated, it is assumed that the grain size is equal before deformation, i.e., it is constant, and the Equation (25) can be changed into:

(26) $$\varepsilon_{0.5}=A_{0.5}{\dot{\varepsilon }}^{m_{0.5}}\exp(\frac{Q_{0.5}}{RT})$$

**Figure 10:**
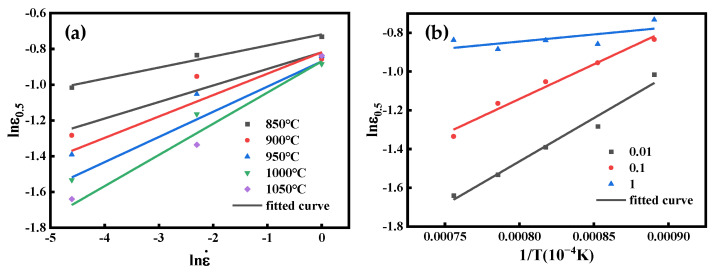
(**a**) Correlation between $\ln\varepsilon_{0.5}$ and $\ln\dot{\varepsilon }$. (**b**) Relationship between $\ln\varepsilon_{0.5}$ and 1/*T*.

The coefficients of the characteristic strain model obtained from Figure 10a,b are $m_{0.5}$ = 0.117778 and $Q_{0.5}$ = 26369.85 J/mol; substituting the parameters into Equation (26), $A_{0.5}$ = 0.032789 can be obtained. Comprehensively, the characteristic strain model is obtained as follows:

(27) $$\varepsilon_{0.5}=0.032789{\dot{\varepsilon }}^{0.117778}\exp(\frac{26369.85}{RT})$$

#### 4.2.4. Establishment of Dynamic Recrystallization Fraction Model for GCr15 Steel Warm–Hot Forming Process

When the strain value reaches 50%, the recrystallization fraction is 0.5, and $k_{d}$ = ln2 = 0.693 can be obtained according to Equation (16).

Equation (16) can be transformed into the following form:

(28) $$\ln[-\ln(1-X_{\mathrm{drx}})]=\ln0.693+n_{\mathrm{drx}}\cdot \ln\left(\frac{\varepsilon -\varepsilon_{c}}{\varepsilon_{0.5}-\varepsilon_{c}}\right)$$

Therefore, it can be concluded that:

(29) $$n_{\mathrm{drx}}=\frac{\partial \ln[-\ln(1-X_{\mathrm{drx}})]}{\partial \ln\left(\frac{\varepsilon -\varepsilon_{c}}{\varepsilon_{0.5}-\varepsilon_{c}}\right)}$$

**Figure 11:**
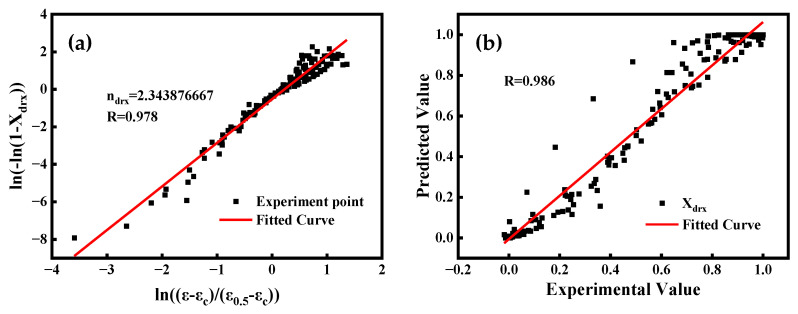
(**a**) Correlation between $\ln[-\ln(1-X_{\mathrm{drx}})]$ and $\ln[(\varepsilon -\varepsilon_{c})/(\varepsilon_{0.5}-\varepsilon_{c})]$. (**b**) The correlation between the predicted value of DRX fraction and the calculated value of GCr15.

The correlation between $\ln[(\varepsilon -\varepsilon_{c})/(\varepsilon_{0.5}-\varepsilon_{c})]$ and $\ln[-\ln(1-X_{\mathrm{drx}})]$ is depicted in Figure 11a, and it is fitted by the least squares method. $n_{\mathrm{drx}}$ can be defined by the slope of the straight line, which is equal to 2.343876667.

In summary, the dynamic recrystallization percentage model is:

(30) $$\left\{\begin{array}{l} X_{\mathrm{drx}}=1-\exp\left[-0.693{\left(\frac{\varepsilon -\varepsilon_{c}}{\varepsilon_{0.5}-\varepsilon_{c}}\right)}^{2.343876667}\right]\\ \varepsilon_{0.5}=0.032789{\dot{\varepsilon }}^{0.117778}\exp(\frac{26369.85}{RT}) \end{array}\right.$$

The Equation (30) can be used to calculate the DRX fraction of GCr15 steel for various deformation conditions. Figure 11b compares the experimental and predicted values of the dynamic recrystallization percentage of GCr15. The predicted results exhibit a favorable level of conformity with the practical outcomes, and the correlation coefficient R [30] is 0.986. Therefore, the dynamic recrystallization kinetic model established above can well predict the dynamic recrystallization of GCr15 steel during the warm–hot deformation process.

## 5. Numerical Simulation of Microstructure Evolution and DRX Model Verification of GCr15 Warm Compression Forming Process

The Hansel–Spittle, dynamic recrystallization, and critical strain models developed for GCr15 steel will be integrated into the Forge^®^ numerical simulation software through secondary development. This will enable the software to perform warm compression simulations to validate the dynamic recrystallization model of GCr15 steel on cylindrical specimens.

Finite element simulation is performed on 1/12 of the cylindrical sample, which is assumed to have cylindrical symmetry, for compression testing. The finite element simulation utilizes the Forge software from Transvalor, a company based in France. To ensure accuracy and convergence in the finite element calculation, tetrahedral elements are used to divide the parts. The upper and lower indenters are meshed with a size of 0.4 mm, while the cylindrical sample is meshed with a size of 0.15 mm. The thermal boundary conditions applied in the simulation are set to adiabatic conditions, similar to those used in the isothermal compression test. Other conditions are set to be consistent with the actual isothermal compression test.

### 5.1. Numerical Simulation of Warm Forming and Compression Process of Cylindrical Specimen

To validate the accuracy of the dynamic recrystallization models developed previously, a comparison was conducted between the numerical simulation results and the actual microstructure results from the compression tests.

**Figure 12:**
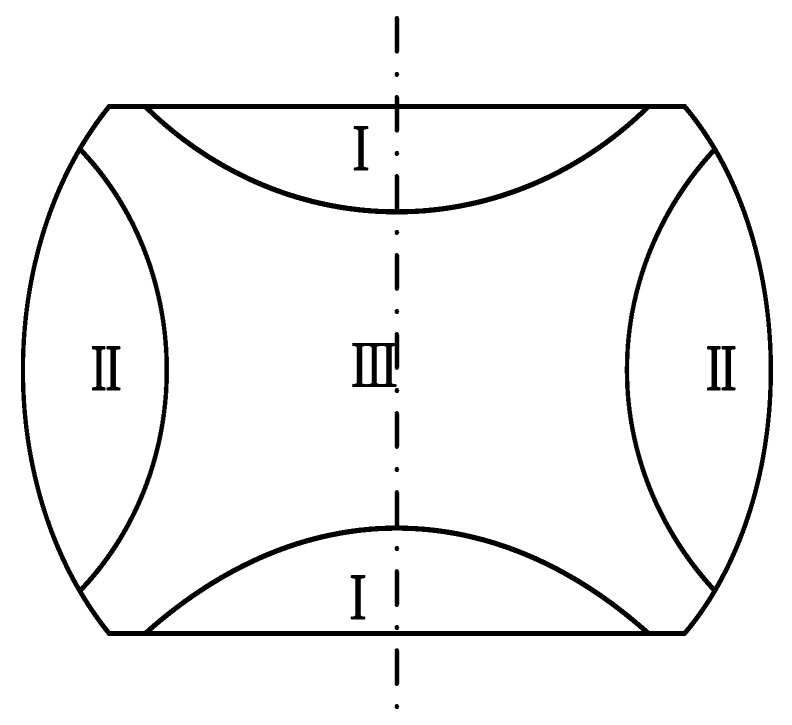
The non-uniformity schematic of the compressed specimen.

Due to friction between the specimen and die surface during the deformation process, the degree of deformation across different regions of the cylindrical sample varies. Figure 12 illustrates that the specimens can be roughly categorized into three distinct regions. Region I is classified as a zone of challenging deformation due to the substantial frictional forces between the upper and lower dies.

Region II is subject to lower frictional forces and shows a more uniform deformation. During the deformation process, it is under the stress state of “two-tensile-one-compress,” resulting in substantial deformation.

Region III is the zone with the largest deformation. Despite the presence of strong three-directional compressive stress, it is located further away from the contact surface and is less obstructed during deformation. Hence, it is the most conducive area for slip and exhibits the greatest amount of deformation.

### 5.2. Simulation Results and Analysis of Deformation of GCr15 Specimen

#### 5.2.1. Dynamic Recrystallization Fraction Result and Analysis

**Figure 13:**
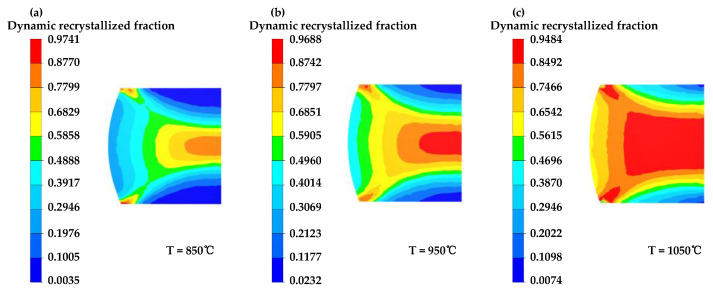
The fraction distribution of dynamic recrystallization of GCr15 steel under various temperature conditions at 50% compression strain and 0.1 s^−1^ strain rate: (**a**) T = 850 °C, (**b**) T = 950 °C, (**c**) T = 1050 °C.

**Figure 14:**
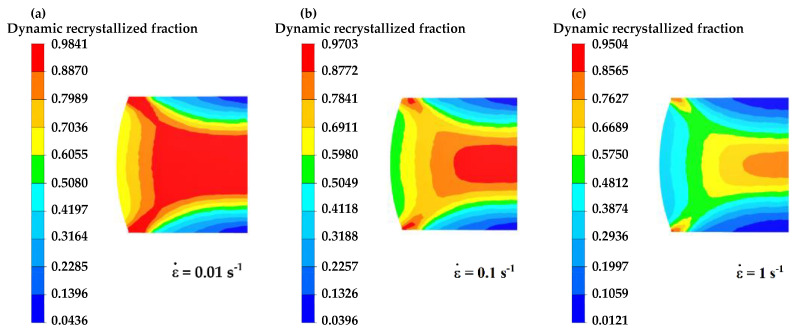
The distribution of dynamic recrystallization percentages at different strain rates at a temperature of 1000 °C and 50% compression strain: (**a**) $\dot{\varepsilon }=\;0.01\;s^{-1}$, (**b**) $\dot{\varepsilon }=\;0.1\;s^{-1}$, (**c**) $\dot{\varepsilon }={\;1\;s}^{-1}$.

**Figure 15:**
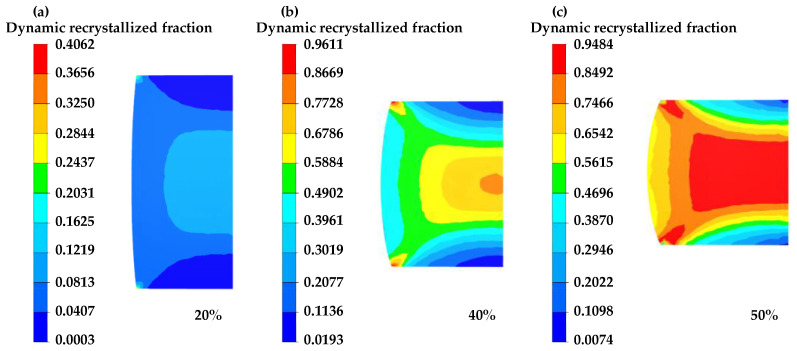
The dynamic recrystallization percentage distribution of different compression strains at 1050 °C/0.1 s^−1^: (**a**) 20%, (**b**) 40%, (**c**) 50%.

Figure 13, Figure 14 and Figure 15 illustrate the simulated results of the DRX fraction of GCr15 steel under varying deformation conditions.

Figure 13 demonstrates that, as the temperature increases, the region of dynamic recrystallization becomes more extensive. The dynamic recrystallization fraction in Region III increases gradually and spreads outwards. This is due to the stronger thermal activation and increased atomic diffusion at higher temperatures, making the material more susceptible to dynamic recrystallization.

According to Figure 14, the extent of dynamic recrystallization decreases with increasing strain rate, leading to a reduction in the degree of recrystallization. This is because, at higher strain rates, deformation occurs more rapidly, increasing the critical strain and making it less likely for the material to undergo dynamic recrystallization internally.

Figure 15 demonstrates that, as the compression strain elevates, the DRX fraction increases, and the region of recrystallization expands. At lower deflection, some areas do not undergo dynamic recrystallization due to the non-uniformity of deformation and not reaching critical strain. However, as the deflection increases, the number of regions reaching the critical strain increases, resulting in an increase in the region and fraction of dynamic recrystallization.

#### 5.2.2. Results and Analysis of Grain Size Distribution of Dynamic Recrystallization during Forming

**Figure 16:**
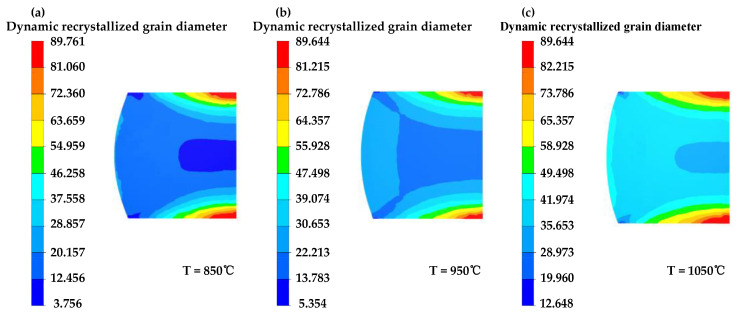
The grain size distribution map for GCr15 steel under different temperature conditions at 50% compression strain and 0.1 s^−1^ strain rate: (**a**) T = 850 °C, (**b**) T = 950 °C, (**c**) T = 1050 °C.

**Figure 17:**
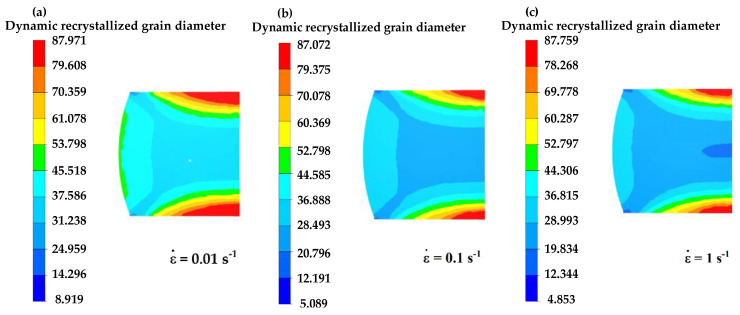
The distribution of grain sizes at different equivalent strain rates at a temperature of 1000 °C and the compression strains of 50%: (**a**) $\dot{\varepsilon }=\;0.01\;s^{-1}$, (**b**) $\dot{\varepsilon }=\;0.1\;s^{-1}$, (**c**) $\dot{\varepsilon }={\;1\;s}^{-1}$.

**Figure 18:**
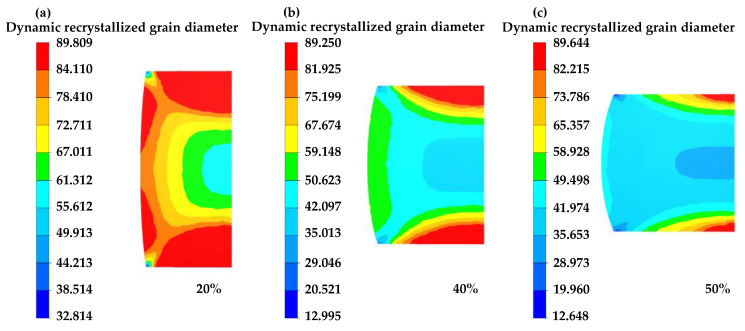
The distribution of grain sizes of different compression strains at 1050 °C/0.1 s^−1^: (**a**) 20%, (**b**) 40%, (**c**) 50%.

Figure 16, Figure 17 and Figure 18 illustrate the distribution of dynamic recrystallization grain size. Grain size elevates with increasing temperature and decreases with increasing equivalent strain rate. An increase in the compression strain leads to a refinement in grain size.

#### 5.2.3. Experimental Verification of Simulation Results for GCr15 Dynamic Recrystallization

**Figure 19:**
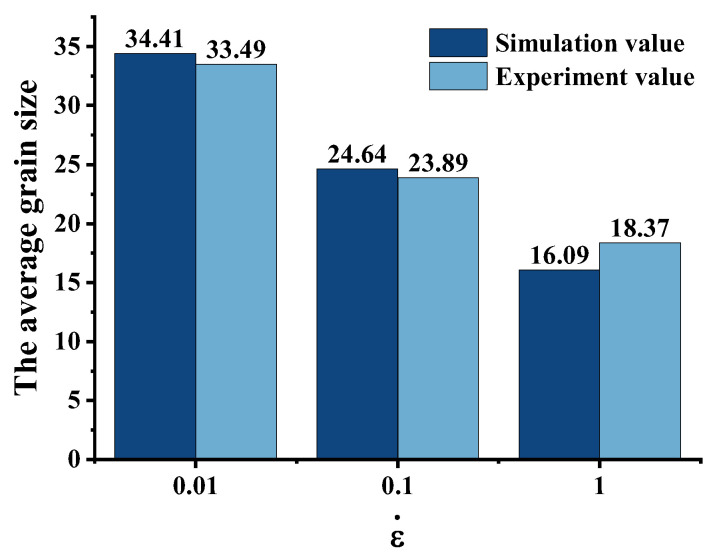
DRX average grain size from simulation and experiment with different equivalent strain rates (1050 °C, 50%).

**Figure 20:**
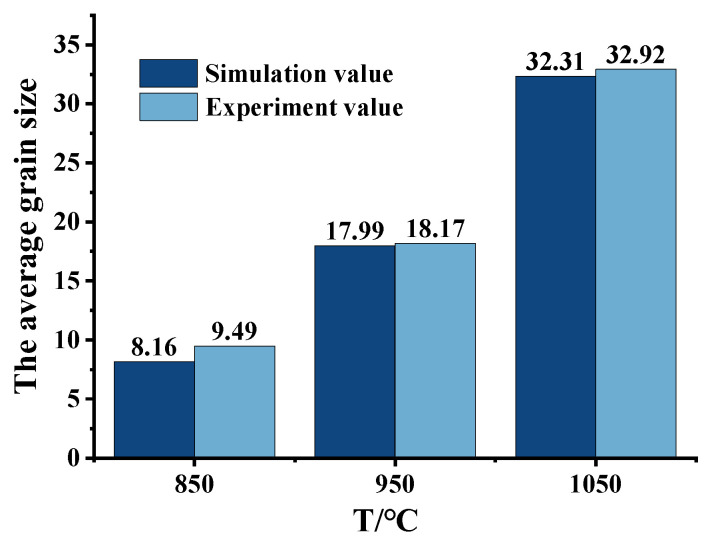
Simulation predicted and experimental DRX average grain size at different temperatures (0.1 s^−1^, 50%).

**Figure 21:**
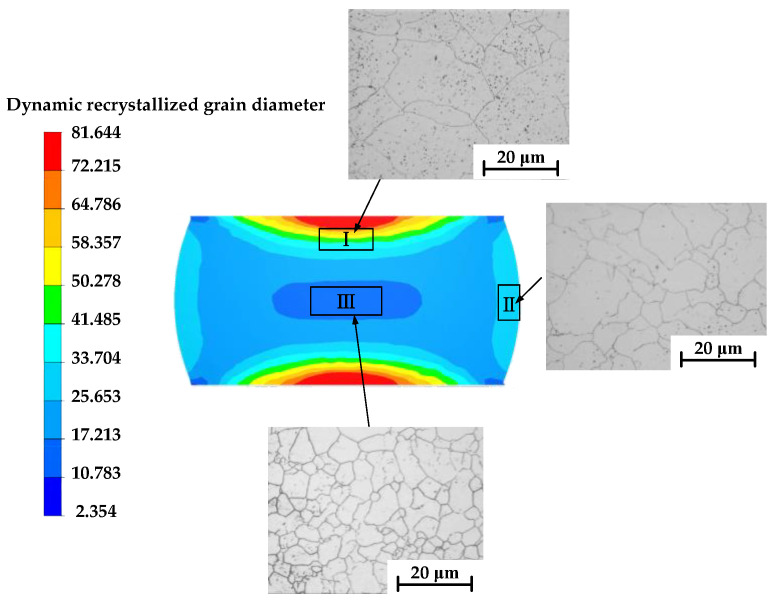
The distribution of grain sizes at 1050 °C/0.1 s^−1^.

Figure 19 and Figure 20 show the comparison between the simulation results of the mean grain size of DRX and the experimental results. At the same time, the relative error between the two was calculated, which was 5.75%. Figure 21 shows the grain size results of each region obtained from the sample upsetting simulation. The high degree of agreement observed between the metallographic images of the corresponding area of the compressed specimens and the simulation results further confirms the accuracy of the established model. This also demonstrates the reliability of using finite element software (Forge^®^ 3.2)to simulate microstructure evolution.

## 6. Conclusions

The Gleeble-1500D testing machine was used in this paper to study the DRX behavior and microstructure evolution of GCr15 bearing steel in the process of warm–hot deformation. The microstructure dynamic recrystallization model and critical strain model of GCr15 under warm–hot deformation conditions were constructed, and the Forge^®^ software was used to validate the developed DRX model’s accuracy. The conclusions can be drawn as follows.

(1) The GCr15 specimens exhibited varying degrees of dynamic recrystallization softening under deformation conditions ranging from 0.01 s^−1^ to 5 s^−1^ and temperatures ranging from 600 °C to 1050 °C. This resulted in a decrease in true stress after attaining the maximum stress. The flow stress curve of the alloy shows three distinct phases. At the beginning of the test, the true stress increases sharply due to the work hardening resulting from the deformation of the specimen. Once the critical strain is reached, the rate of stress increase slows down because of the DRV and DRX mechanisms until it reaches the maximum stress. As the strain level rises, the true stress value declines until it reaches a steady-state stress level, where it stabilizes. On the other hand, at a specific strain rate, the maximum stress decreases as the deformation temperature elevates. Conversely, at a specific temperature, the maximum stress is proportional to the strain rate.
(2) The critical strain model of DRX during the warm–hot forming process of GCr15 was established using the work hardening rate method. The DRX kinetic model of GCr15 steel was developed with the stress–strain method. Under a fixed strain rate, the required strain to achieve the same DRX fraction decreases as the deformation temperature increases. However, at a constant temperature, it increases with an increase in strain rate.
(3) By performing secondary development on the Forge^®^ software, the established deformation dynamic recrystallization model, critical strain model, and Hansel–Spittle constitutive model for GCr15 steel were integrated into the software. The finite element simulation results suggest the distribution of the DRX fraction during the deformation of GCr15. The recrystallization fraction rises with a lower strain rate and a higher deformation temperature, which is completely consistent with the evolution law of the actual DRX volume fraction. In addition, a higher strain rate and a lower temperature can obtain a finer recrystallization grain structure.
(4) The compressed specimens after isothermal compression present a bulging shape, and three typical deformation regions can be observed when the sample is cut along the axial direction. In the severe deformation region of the compressed sample, the deformation resistance is lower, resulting in a higher deformation amount and finer, more uniform grain size. The slight deformation region undergoes relatively uniform deformation due to minimal frictional effects. However, in the difficult deformation region, significant frictional effects make deformation more challenging, resulting in larger grain sizes in this area. Comparing the metallographic photos of the simulation results and the actual test results, it is found that the two have a high degree of agreement. It is further verified that the established related models have high accuracy and can provide a specific theoretical reference for actual production. It also shows that the finite element method (FEM) can provide an effective way for analyzing and predicting the microstructure evolution of the ring warm–hot deformation process.

## Figures and Tables

**Table 1:** The elemental makeup of GCr15 (wt.%).

Fe	C	S	P	Mn	Si	Cr	Mo	Cu	Ni
Balance	0.96	0.006	0.013	0.36	0.19	1.46	0.02	0.06	0.08

**Table 2:** Constitutive equation parameters for GCr15.

*A*	$m_{1}$	$m_{2}$	$m_{3}$	$m_{4}$	$m_{5}$	$m_{7}$	$m_{8}$	$m_{9}$
4.16 × 10^6^	−0.00463	0.1382	−0.093	0.00209	0.00317	−2.0621	2.66 × 10^−4^	−0.8565

**Table 3:** The fitting equation parameters of lnθ-ε at different deformation conditions.

$\dot{\varepsilon }$/s^−1^	T/°C	Fitting Parameters					$\varepsilon_{c}$	$\varepsilon_{p}$
		*A*	*B*	*C*	*D*	*R^2^*		
0.01	850	8.47014	−76.891	740.9507	−2696.4	0.96842	0.091598	0.195
	900	7.71935	−69.0227	850.89	−4010.29	0.97922	0.070726	0.158
	950	8.25778	−121.652	1844.788	−9544.48	0.92666	0.064428	0.133
	1000	7.83118	−129.471	2411.967	−15586.7	0.90924	0.051582	0.111
	1050	8.15413	−168.286	3375.513	−23384.8	0.92356	0.048116	0.094
0.1	850	8.41727	−43.066	248.1561	−664.752	0.95863	0.124435	0.256
	900	7.82255	−46.4115	366.5169	−1184.33	0.96725	0.103157	0.234
	950	8.27077	−73.5862	706.0572	−2532.06	0.97569	0.092949	0.198
	1000	7.86564	−80.9397	902.8293	−3586.9	0.94904	0.083901	0.18
	1050	7.80172	−103.649	1425.969	−6598.36	0.93148	0.072037	0.154
1	850	8.52139	−41.3525	211.2415	−430.082	0.96166	0.163722	0.347
	900	8.38092	−46.3749	258.0802	−561.636	0.96158	0.153172	0.326
	950	7.77295	−39.1519	230.4801	−559.416	0.97828	0.137334	0.303
	1000	7.68793	−45.0763	328.8014	−936.054	0.96539	0.117088	0.266
	1050	7.8954	−55.4391	390.8831	−1030.36	0.9636	0.126455	0.269

**Table 4:** The $\sigma_{\mathrm{sat}}$ of DRV at different deformation conditions.

$\sigma_{\mathrm{sat}}$	0.01 s^−1^	0.1 s^−1^	1 s^−1^
850 °C	136.7623	188.5939	261.4116
900 °C	107.0416	151.9632	204.7171
950 °C	86.7559	120.7993	173.3112
1000 °C	64.4685	94.79529	141.7204
1050 °C	50.6158	81.09207	119.1046

**Table 5:** Characteristic strains under different forming conditions.

	0.01 s^−1^	0.1 s^−1^	1 s^−1^
850 °C	0.362	0.434	0.481
900 °C	0.277	0.385	0.424
950 °C	0.249	0.349	0.432
1000 °C	0.216	0.312	0.413
1050 °C	0.194	0.263	0.433

## Data Availability

The data presented in this study are available on request from the corresponding author. The data are not publicly available due to these data being part of ongoing research.
